# Evaluation of Iron Deficiency Anemia Frequency as a Risk Factor in Glaucoma

**DOI:** 10.1155/2018/1456323

**Published:** 2018-08-01

**Authors:** Penpe Gul Firat, Ersan Ersin Demirel, Seyhan Dikci, Irfan Kuku, Oguzhan Genc

**Affiliations:** ^1^Associate Professor, Inonu University School of Medicine, Department of Ophthalmology, Malatya, Turkey; ^2^MD, Sarkısla State Hospital, Department of Ophthalmology, Sivas, Turkey; ^3^Assistant Professor, Inonu University School of Medicine, Department of Ophthalmology, Malatya, Turkey; ^4^Professor, Inonu University School of Medicine, Department of Hematology, Malatya, Turkey; ^5^MD, Mujde Hospital, Department of Ophthalmology, Malatya, Turkey

## Abstract

**Purpose:**

Iron deficiency anemia is an important public health problem. Also it is considered to be a risk factor for many diseases. The study demonstrates the iron deficiency anemia frequency in glaucoma patients and compares with the normal subjects. We aimed to determine the iron deficiency anemia frequency in glaucoma patients.

**Methods:**

Prospective, controlled study in a single university hospital setting. A total of 130 normal subjects (Group 1) and 131 glaucoma patients (Group 2) were included. The erythrocytes parameters, hemoglobin, red blood cell, hematocrit, mean corpuscular volume, mean corpuscular hemoglobin concentration, mean corpuscular hemoglobin, and red blood cell distribution width, and iron status indicators, Fe (iron), total iron binding capacity, and ferritin of the cases, in normal subjects and glaucoma patients were compared.

**Results:**

There was no statistically significant difference for the erythrocyte parameters between the groups (p≥0.05). The number of the patients with iron deficiency anemia in both groups was similar. No statistically significant difference was found in the comparison of erythrocyte parameters and iron status indicators values according to the number of antiglaucomatous agents and visual field changes according to the presence of anemia in Group 2 (p≥0.05). A statistically significant difference was found only in MCH when the erythrocyte parameters and iron status indicators values of the cases in glaucoma patients were compared with the glaucoma duration (p<0.05).

**Conclusion:**

The iron deficiency anemia frequency was like the normal population in glaucoma patients.

## 1. Introduction

Glaucoma is a chronic neuropathy that leads to progressive atrophy in the optic nerve head, degeneration in retinal ganglion cells, and visual field losses and causes visual loss due to optic atrophy when not treated. These changes usually exist together with intraocular pressure elevation (IOP) [1]. Increased IOP is not the only factor in glaucoma. Some publications in recent years have focused on the role of oxidative stress in the pathogenesis of glaucoma [2]. Oxidative stress can be defined as an imbalance between the high level of the intracellular concentrations of reactive oxygen species (ROS) in physiologic values while antioxidant level being insufficient. This imbalance has been shown to cause retinal ganglion cell death [3].

Anemia is clinically defined as a blood hemoglobin or hematocrit value under the valid reference range for the patient [4]. Iron deficiency is the most common form of anemia. It was demonstrated that oxidants were elevated, and the antioxidants were decreased in iron deficiency anemia (IDA) [5]. Besides iron is a vital element for myelin production [6]. Lack of iron is associated with hypomyelination which can affect all of the nervous systems, as well as the optic nerve, which is constituted mainly by myelinated fibers [7]. These findings highlight the importance of investigating the role of IDA on glaucoma. To date, the effects of IDA which can affect the oxidant– antioxidant system on glaucoma have not been studied.

The aim of this study was to determine the IDA frequency in glaucoma patients which is a neurodegenerative disease. We compared the IDA incidence in the normal population and the glaucoma patients to evaluate whether detecting and treating the anemia could slow down or stop the progress of the disorder.

## 2. Material and Method

A total of 131 glaucoma patients who were being followed up at our Glaucoma Unit and met the study inclusion criteria and 130 healthy individuals were included in the study. The healthy individuals were included in Group 1 and the patients with glaucoma in Group 2.

The study was conducted in accordance with the ethical standards stated in the Declaration of Helsinki and approved by our institutional ethics board. The patients were informed on the purpose of the study and the procedures to be performed in detail and consent was obtained.

Each subject underwent a full ophthalmic examination, including best-corrected visual acuity, IOP measurement with a Goldmann applanation tonometry, slit-lamp biomicroscopy, gonioscopy, stereoscopic fundus evaluation on the slit lamp using a 90-diopter lens, and visual field examination with Humphrey Field Analyzer (HFA) (Humphrey-Zeiss Systems, Dublin, CA, USA) Swedish Interactive Threshold Algorithm (SITA) 30–2 test. Each test was performed on the same machine with the same technician. The tests were repeated in the same week if an abnormality was found in the reliability indexes (fixation loss >20%, false positive response >25%, false negative response >25%). Both test strategies were investigated in terms of test duration and mean deviation (MD).

Inclusion criteria in normal subjects were IOP under 21 mmHg, normal optic disc, and visual field examination and the lack of any systemic disease or systemic drug use. Inclusion criteria in glaucoma patients were a minimum of one-year follow-up at our clinic, the presence of glaucomatous damage in the optic disc (glaucomatous cupping and/or glaucomatous optic nerve head changes), the use of at least one antiglaucomatous agent, and presence of glaucomatous visual field changes in a minimum of two computerized visual field examinations (30-2 SITA). History of laser treatment or trauma, presence of cornea or lens pathology, presence of uveitis, posterior segment pathology, neurodegenerative diseases of the central nervous system, and systemic diseases such as diabetes that can predispose to such disorders were accepted as exclusion criteria for both groups.

Erythrocyte parameters were studied with the Beckman Coulter LH 780, biochemical data, Fe and IBC with the Abbott Architect c16000, and Ferritin with the Siemens immulite 2000 device. IDA was defined as hemoglobin concentration <12.0 g/dL and serum ferritin concentration <15.0 *μ*g/L [8].

Quantitative data were presented as mean ± standard deviation (SD) or median (min-max). Compliance with a normal distribution was determined with the Shapiro-Wilk test. The homogeneity of the variances was evaluated with the Levene test. One-way variance analysis and the Kruskal-Wallis test were used for statistical analyses and the* t*-test and Mann–Whitney U test were used for independent samples. When the one-way variance analysis result was important, multiple comparisons were made with the Tukey test. The anemia incidence of the subgroups in glaucoma was compared with Pearson Chi-Square test. A p value <0.05 was accepted as statistically significant. The study was powered to detect a hemoglobin concentration difference of 0.35 gr/dlt with a *β* error of 0.80 and an *α* error of 0.05 in a 2-tailed test.

## 3. Results

Patients who fulfilled the entry criteria were enrolled in this prospective, controlled study. There were 130 normal subjects (63 females, 67 males) in control group and 131 glaucoma patients (74 females, 57 males). The mean age of the normal subjects was 50,01 ± 13,6 years; the mean age of the glaucoma patients was 49,01 ± 10,7 years. There were no statistically significant differences in age (p=0.424) and gender (p=0.194) among the groups.

There were no statistically significant differences in the erythrocytes parameters and the iron status indicator values between the groups. (Table 1)

We detected 26 IDA patients in normal subjects and 24 patients with IDA in glaucoma group. No statistically significant difference was observed between the groups in terms of the number of patients with IDA (p=0.48, p>0.05, respectively).

The glaucoma group was consisting of primer open angle patients glaucoma (POAG) (n=57), pseudoexfoliation glaucoma (PEXG) patients (n=43), and normotensive glaucoma (NTG) patients (n=31). The number of patients with IDA was fourteen in POAG, five in PEXG, and seven in NTG patients. No statistically significant difference was found between the glaucoma subgroups regarding the IDA incidence (p=0.251, p>0.05).

No statistically significant difference was found regarding the number of antiglaucomatous agents used between the patients with and without IDA in glaucoma patients (p=0.68, p>0.05). Similarly, changes in the mean deviation (MD) in the right and left eye 30-2 computerized visual fields obtained at intervals of at least 6 months in these patients were not found to be statistically significant (p=0.44, p=0.21, p>0.05, respectively) (Table 2).

No statistically significant difference was found between the erythrocyte parameters and the iron status indicator values of the patients in glaucoma patients according to the number of antiglaucomatous agents used (Table 3).

There was no statistically significant difference between the erythrocytes parameters and the iron status indicator values according to the duration of glaucoma in glaucoma patients, but the difference in MCH values was statistically significant (p=0.03, p<0.05) (Table 4).

## 4. Discussion

This study was undertaken to determine whether the IDA frequency is higher in glaucoma patients than the normal population. We could not find any statistically significant difference between the groups according to the IDA frequency. Although ocular findings of severe anemia have been defined more commonly in recent years, we could not find any study investigating the relationship between glaucoma and IDA. The severity of retinal findings is consistent with the severity of the anemia. Retinal ischemia findings of optic disc edema, retinal hemorrhages, and soft exudes can be seen in certain patients with anemia [9]. Venous congestion, retinal hemorrhages, and soft exudes can similarly also be seen in retinal vein occlusion and anterior ischemic optic neuropathy [10]. Although the pathophysiology is unknown, it is likely associated with retinal hypoxia [11].

Considering that IDA may accelerate the visual field loss in patients with glaucoma, we obtained two separate 30-2 visual field tests with an interval of six months in patients with and without iron deficiency anemia but the difference in mean deviations in visual field tests was not found to be statistically significant. We also showed that the need for antiglaucomatous agents also did not increase in glaucoma patients with IDA. A possible explanation can be the severity of anemia in our study. The range of hemoglobin level in anemia patients was 9-12 g/dl in normal group and 9.2-12 g/dl in glaucoma group. As shown in previous reports ocular side effects of anemia were seen in severe anemia cases. Retinal findings can be seen when the hemoglobin levels were less than 6 g/dl [12]. Our patients' anemia can be classified as mild and moderate according to the WHO anemia classification [13]. Carraro et al. demonstrated the prevalence of retinopathy in patients with anemia or thrombocytopenia. They showed that retinopathy was closely associated with the severity of the anemia. But in their study acute blood loss was the main cause of the retinopathy and anemia not IDA [14].

We also demonstrated that the anemia frequencies in POAG, NTG, and PEXG are the same. Goldberg et al. studied the systemic factors including hemoglobin concentration and hematocrit levels in NTG and ocular hypertension patients. Similar to our study they found out that the hemoglobin and hematocrit levels were the same in NTG and ocular hypertension patients [15].

Iron is an essential element for many functions in the cell like oxygen transport, myelin synthesis, and oxidative phosphorylation. On the other hand if the iron is found in excess amount it can cause reactive oxygen species formation that can cause cellular degeneration and damage [16]. Disorders in iron metabolism have been shown to cause many neurodegenerative diseases such as Parkinson's disease, Alzheimer's disease, Huntington's disease, Hallervorden-Spatz disease, and Friedreich disease [17, 18]. Since glaucoma is also considered as a neurodegenerative disorder, IDA frequency in patients with glaucoma was compared with normal individuals and no statistically significant difference was found between the two groups in our study.

However, the neuronal affinity of iron and ferritin may increase in glaucoma patients. Considering that the serum iron and ferritin level may not be correlated with the brain iron and ferritin levels, we believe that detailed neuropathology studies investigating brain iron and ferritin levels in glaucoma patients are required.

We therefore concluded that it was not necessary to change the frequency of follow-ups and number of antiglaucomatous agents in glaucoma patients who are found to have IDA.

The strengths of this study are that it is a prospective, controlled study. Also, to the best of our knowledge, this is the first study that demonstrates the IDA frequency in glaucoma. Limitation of our study is that the study includes mild and moderate anemia patients. Large sample sized studies with severe anemia patients are needed to better understand the effect of anemia on glaucoma.

## Figures and Tables

**Table 1:** Comparisonof Fe, TIBC and ferritin valuesbetween Group 1 and Group 2.

**Variables (mean±sd)**	**Group 1**	** Group 2**	**P**^**∗**^
**Hb **(g/dl)	13.73±1.8	13.48±1.7	0.21
**Rbc **(10^6^/ml)	4.75±0.80	4.68±0.79	0.51
**Htc **(%)	40.94±5.29	40.41±4.93	0.40
**MCV **(fl)	87.20±7.87	87.62±7.19	0.62
**MCHC **(g/dl)	33.13±2.12	32.90±1.40	0.30
**MCH (**pg**)**	29.35±3.26	28.97±3.52	0.35
**RDW **(%)	14.29±1.81	13.81±2.28	0.06
**Fe (** *µ*g/dl)	74.38±36.2	71.82±33.89	0.55
**TIBC **(*µ*g/dl**)**	269.67±64.15	273.12±66.26	0.69
**Ferritin **(n/dl), [Median (Min-Max)]	70.60 (1-240)	70.07(2-294))	0.95
**Plt **10^3^/ml	241.22±80.82	268.39±72.00	**0.0**4^*∗*^

**Hb:** hemoglobin; **Rbc: red blood cells**; **Htc: **hematocrit;** MCV: mean corpuscular volume**;** MCHC:** mean corpuscular hemoglobin concentration; **MCH: mean corpuscular hemoglobin**;** RDW: **red blood cell distribution width; **Fe:** iron; **TIBC:** Total iron binding capacity; **Plt:** platelet;** sd: **standard deviation; **Min:** minimum; **Max:** maximum.

**Table 2:** Comparisonofthenumber of antiglaucomatous agents used and changes of visual fields patients in glaucoma patients according to presence of anemia.

	Patients With Anemia (Mean ± SD)	Patients Without Anemia (Mean ± SD)	P value
The number of antiglaucomatous agents used	2.36±0.7	2.85±1.3	0.68
MD Right eye	0.08±1.9	-0.55±2.6	0.44
MD Left eye	-2.21±4.3	-0.24±1.5	0.21

**SD:** standard deviation**; MD Right-Left:** changes in the mean deviation in the 2 separate visual fields with 6 monthly intervals.

**Table 3:** Comparisonthe hemogram, iron, TIBC, and ferritin valuesaccording to thenumber of antiglaucomatous agents used in Group 2.

**The number of antiglaucomatous agents used** **The number of patients**	**1** (Mean± SD) n=28	**2** (Mean ± SD) n=31	**3** (Mean ± SD) n=36	**4** (Mean ± SD) n=36	P value
**Hb** g/dl	13.48±1.50	12.85±1.90	13.45±2.04	14.11±1.54	0.14
**Rbc** 10^6^/ml	4.87±0.55	4.46±0.45	5.00 ±1.73	4.83±0.46	0.28
**Htc** %	39.66±4.48	38.71±5.67	39.68±6.43	42.11±3.25	0.16
**MCV** fl	83.69±4.53	86.66±6.84	88.07±10.27	88.04±6.56	0.34
**MCHC** g/dl	33.25±2.09	32.77±1.00	32.56±1.76	32.87±1.32	0.65
**MCH** pg	28.40±2.15	28.85±2.40	29.52±3.87	27.48±6.02	0.69
**RDW** %	13.38±1.33	14.02 ±1.60	13.72±1.43	13.39±1.06	0.39
**Fe** *µ*g/dl	69.69±31.24	74.42±32.25	75.44±42.62	84.00±41.39	0.71
**TDBK** *µ*g/dl	293.07±65.82	284.95±80.11	261.22±66.25	253.81±52.5	0.25
**Ferritin** ng/ml Median (Min-Max)	69.90 (5-250)	67.46 (3-254)	67.58 (3-238)	70.88 (12-277)	0.93
**Plt **10^3^/ml	230.77±50.99	287.79±85.93	257.11±38.90	264.67±55.28	0,07

**Hb:** hemoglobin; **Rbc: red blood cells**; **Htc: Hematocrit**;** MCV: mean corpuscular volume**;** MCHC:** mean corpuscular hemoglobin concentration; **MCH: mean corpuscular hemoglobin**;** RDW: **red blood cell distribution width; **Fe:** iron; **TIBC:** total iron binding capacity;** SD: **standard deviation; **Min:** minimum; **Max:** maximum.

**Table 4:** Comparison of the hemogram, iron, TIBC, and ferritin values according to the duration of glaucoma in Group 2.

**Duration of Glaucoma (mean±sd)**	**3 years**	**4 years**	**5 years**	**6 years and over**	**P value**
**Hb **g/dl	12.71±2.03	13.36±1.73	13.45±1.68	14.09±1.70	0.13
**Rbc **10^6^/ml	4.72±1.24	4.63±0.62	4.98±1.11	4.67±0.64	0.64
**Htc **%	37.72±5.50	40.43±5.31	40.32±5.32	41.30±4.52	0.18
**MCV fl**	84.23±7.41	85.56±8.00	86.38±6.42	90.30±7.30	0.058
**MCHC g/dl**	32.54±1.45	32.58±1.53	32.95±1.83	33.10±1.28	0.59
**MCH pg**	28.35±2.89	26.48±6.83	28.47±2.34	30.27±2.6	**0.0**3^**∗**^
**RDW %**	14.29±1.90	13.69±1.40	13.56±1.18	13.27±0.86	0.14
**Fe ** *µ*g/dl	75.45±31.34	71.07±30.93	76.05±36.65	81.32±45.97	0.87
**TDBK** *µ*g/dl	277.88±59.48	286.86±47.19	281.86±73.99	247.86±77.68	0.25
**Ferritin n/dl.** [Median (Min-Maks)]	21 (3-250)	26 (3-250)	58 (5-183)	71 (13-254)	0.14

**Hb:** hemoglobin; **Rbc: red blood cells, Htc: hematocrit**;** MCV: mean corpuscular volume**;** MCHC:** mean corpuscular hemoglobin concentration; **MCH: mean corpuscular hemoglobin**;** RDW: **red blood cell distribution width; **Fe:** iron; **TIBC:** total iron binding capacity;** sd: **Standart deviation; **Min:** minimum; **Max:** maximum.

## Data Availability

The data used to support the findings of this study are available from the corresponding author upon request.
